# Vimentin immunization induces TH2/TH17 cell activation and autoantibody production in a novel mouse model of bleomycin induced systemic sclerosis

**DOI:** 10.3389/fimmu.2025.1639601

**Published:** 2025-11-07

**Authors:** Chae Rim Lee, Seon-Yeong Lee, Jeonghyeon Moon, Jae-Deog Kim, Su Been Jeon, Kun Hee Lee, Tae Ho Kim, Jeong Won Choi, Sang-Uk Seo, Mi-La Cho

**Affiliations:** 1 Lab of Translational ImmunoMedicine, Catholic Research Institute of Medical Science, College of Medicine, The Catholic University of Korea, Seoul, Republic of Korea; 2 Department of Pathology, College of Medicine, The Catholic University of Korea, Seoul, Republic of Korea; 3 Department of Biomedicine and Health Sciences, College of Medicine, The Catholic University of Korea, Seoul, Republic of Korea; 4 Department of Life Science in Dentistry, School of Dentistry, Dental and Life Science Institute, Pusan National University, Yangsan, Republic of Korea; 5 Education and Research Team for Life Science on Dentistry, Pusan National University, Yangsan, Republic of Korea; 6 Department of Microbiology, College of Medicine, The Catholic University of Korea, Seoul, Republic of Korea

**Keywords:** systemic sclerosis, vimentin immunization, autoimmunity, fibrosis, Th17

## Abstract

**Introduction:**

Systemic sclerosis (SSc) is an autoimmune disease characterized by progressive fibrosis of the skin and lung tissues. Currently available drugs delay SSc progression but do not reverse the sclerotic lesions or cure the disease. Studies of its pathogenesis have been hindered by the lack of an appropriate animal model, as the widely used mouse model of bleomycin (BLM)-induced scleroderma does not reproduce the immune cell activation seen in humans.

**Methods:**

Here we describe an improved mouse model of SSc, achieved by combining BLM administration with immunization against the structural protein vimentin, injected in homogenized form with complete Freund’s adjuvant (CFA).

**Results:**

An examination of the immune cell modifications and pathological changes in skin and lung showed both more severe fibrosis and an increase in systemic Th2 and Th17 cells and autoantibodies compared to mice treated with BLM alone. The levels of pro-inflammatory cytokines in the lesions of vimentin-immunized mice were also significantly increased.

**Discussion:**

This improved model system offers dual advantages for advancing SSc research: it enables deeper mechanistic investigation of the interconnected pathways driving both fibrosis and autoimmune activation, while simultaneously providing a more relevant preclinical platform for evaluating novel therapeutic strategies targeting multiple disease components.

## 1 Introduction

Systemic sclerosis (SSc, scleroderma) is an autoimmune-driven connective tissue disease characterized by progressive tissue fibrosis that particularly affects the skin and lungs (1, 2). Unlike other autoimmune diseases, SSc causes tremendous suffering and premature death (3). The etiology of the disease remains poorly understood, and current treatment strategies delay SSc but do not halt or reverse the fibrosis (4–6).

An abnormal immune response is a key factor in the pathogenesis of SSc (7–9) and accumulations of T cells, macrophage, monocytes, dendritic cells, and other immune cells are found in SSc lesions (10). The presence of activated T cells and macrophages suggests their critical role in SSc progression, while activated B cells account for autoantibody production (11–13). In particular, Th17 cells, which mainly produce IL-17, occur with a high frequency not only in the lesion but also in the peripheral blood of SSc patients (14, 15). TGF-β, IL-23, and IL-6, which promote Th17 differentiation, are also present at high levels in the blood of SSc patients (15–17).

The widely-used bleomycin (BLM)-induced mouse model mimics the dermal and pulmonary fibrosis that characterizes SSc in humans (18–20). However, although the BLM model replicates some signaling patterns of SSc, it lacks important features, particularly autoreactive B cell activation (21). This limitation has hindered the development of effective treatments for this disease.

Vimentin, a major intermediate filament protein in dermal fibroblasts, is essential for cell motility, migration, and wound healing. Growing evidence indicates its significant role in systemic sclerosis (SSc) pathogenesis through autoimmune mechanisms (22, 23). Early diffuse cutaneous systemic sclerosis (dSSc) is characterized by elevated serum citrullinated vimentin (VICM) levels, which reflect disease activity and progression rate, with the highest concentrations seen in patients with rapid progression compared to healthy controls (24). Histological examination reveals high vimentin expression in endothelial cells of SSc patients, with electron microscopy showing swollen endothelial cells filled with vimentin filaments in occluded microvessels (25).

Based on this evidence, we reasoned that vimentin could serve as an ideal target for improving SSc animal models. The present study aimed to test whether combining bleomycin administration with vimentin immunization would create a more clinically relevant model incorporating both fibrotic and autoimmune components of human SSc.

## 2 Materials and methods

### 2.1 Synthesis of vimentin protein

The pET28a plasmid (Addgene), which contains the T7 promoter and terminator, was used to obtain a Vim-expressing vector. The gene encoding mouse Vim (GenBank accession number: NP_035831.2) but with modified codon usage was optimized for expression in *Escherichia coli* (Cosmogenetech). The insert was amplified using a primer set that added a 6×His tag sequence immediately upstream of the Vim gene, allowing the expressed protein to be fused with a 6×His tag at the N-terminus. Subsequently, both the plasmid and the insert were cleaved with NcoI and XhoI restriction enzymes and then ligated together. The expression vector was transformed into BL21 (DE3) competent *E*. *coli* (Enzynomics, Chungcheongnam-do, Korea), which then was cultured at 37°C until the optical density at 600 nm reached 0.6–0.8. Following the addition of 0.2 mM isopropyl β-D-1-thiogalactopyranoside to induce Vim expression, the culture was incubated for an additional 4 h. Subsequently, the cells were harvested, resuspended in lysis buffer (50 mM Tris-HCl, 300 mM NaCl, pH 7.5), and lysed via sonication. Then the total lysate was centrifuged to separate insoluble and soluble proteins in the pellet and supernatant, respectively. The pellet was resuspended in the original volume of the total lysate. Vim expression was confirmed by SDS-PAGE, followed by Western blotting using the primary antibody anti-6×His tag (MA1–135) (Thermo Fisher Scientific, MA, USA) and the secondary antibody anti-rabbit IgG-HRP (A90–116P) (Bethyl Laboratories, TX, USA). Vim was isolated by resuspending the harvested cells in wash buffer (50 mM Tris-HCl, 300 mM NaCl, 20 mM imidazole, 6 M urea, pH 7.5) and then purifying the protein using Ni-NTA resin (Qiagen, California, USA) followed by dialysis against 5 mM HCl. The purity of the obtained Vim was analyzed using the ‘plot lanes’ option in ImageJ software.

### 2.2 Animals and induction of systemic sclerosis

Six-week-old male BALB/c mice (Orient Bio; n=12, housed 5 per cage) were maintained in polysulfon Individually ventilated cage in a specific-pathogen-free system. All animal experimental procedures were reviewed and approved by Department of Laboratory Animals, Institutional Animal Care and Use Committee (IACUC) of the School of Medicine, the Catholic University of Korea (IACUC No. 2023–0030–05).

To induce SSc, the mice were initially immunized with 50 µg Vim protein (Department of Microbiology, The Catholic University of Korea) and complete Freund’s adjuvant (CFA; Chondrex) via intradermal injection at two sites on their backs. To provide continuous analgesia after intradermal injections, mice were first anesthetized under inhalational isoflurane (3–4% induction, 1.5–2% maintenance via nose cone). Immediately after anesthesia, a single subcutaneous injection of tramadol (25 mg/kg) was administered. Following recovery, acetaminophen was supplied in the drinking water at a concentration of 0.2 mg/mL (26), beginning one hour after tramadol injection and continued for 24 hours (27). All veterinary treatments were performed under the guidance of the attending veterinarian. Subsequently, the mice received daily subcutaneous injections of 50 µg BLM (HY-17565A, MedChemExpress) dissolved in 100 µL phosphate-buffered saline (CBP3071, Dynebio) for 3 weeks. The BLM was injected subcutaneously near the neck region. Two weeks after the first immunization, the mice were immunized again with Vim and incomplete Freund’s adjuvant (Chondrex) for boosting. Control mice consisted of those injected with BLM, either alone or in combination with CFA.

### 2.3 Flow cytometry

Five weeks after the first immunization, blood was collected from mice by retro-orbital venous plexus puncture at the time of sacrifice, and spleens were harvested aseptically. Red blood cells (RBCs) in the blood samples were lysed using ACK lysis buffer according to the manufacturer’s protocol, and the remaining leukocytes were collected for further analysis. Spleen were gently dissociated using sterilized glass slides with frosted ends to create a single-cell suspension. The cell suspension was then treated with ACK lysis buffer to remove residual RBCs and filtered through a 40 μm cell strainer to obtain a single-cell suspension of splenocytes. The cells were washed and resuspended in complete RPMI 1640 medium containing 5% FBS. Cells were then stained with peridinin-chlorophyll protein (PerCP)-conjugated anti-mouse CD4 antibody (Invitrogen) for 30 min at 4°C. Then the cells were permeabilized, fixed using the CytoPerm/CytoFix kit (BD Biosciences) in accordance with the manufacturer’s protocol, and stained with phycoerythrin (PE)-conjugated anti-mouse IL-4 (BD Pharmingen) and fluorescein isothiocyanate (FITC)-conjugated anti-mouse IL-17 (Invitrogen) antibodies. All samples were analyzed using a cytoFLEX flow cytometer (Beckman Coulter). The flow cytometry data were processed using FlowJo™ software.

### 2.4 Immunofluorescence imaging

Helper T cells were detected by staining spleen and lung tissue sections 7 µm thick with FITC-conjugated anti-mouse CD4 (BD Biosciences), Alexa Fluor 647-conjugated anti-mouse IL-4 (BD Biosciences) and PE-conjugated anti-mouse IL-17 (BD Biosciences) antibodies. Anti-Vim antibody-producing B cells were identified by staining splenic tissue sections 5 µm thick with labeled Vim, prepared using the Lightning‐Link FITC antibody labeling kit (Odenton) following the manufacturer’s instructions (Innova Bioscience). The tissue sections were also stained with PE-conjugated anti-mouse CD19 (BD Biosciences) and Alexa Fluor 647-conjugated anti-mouse B220 (BD Biosciences) antibodies. The stained sections were analyzed using a Zeiss LSM 510 Meta confocal microscope (Carl Zeiss).

### 2.5 Enzyme-linked immunosorbent assay

Vim-specific total IgG and IgG1 levels in serum were measured via ELISA. Microtiter plates were coated with Vim (5 μg/mL) and incubated at 4°C overnight, followed by a blocking step for 30 min at room temperature. Each serum sample diluted 1:500 in Tris buffered saline (pH 8.0) was added to the microtiter plates for 1 h, after which time the plates were washed three times. Vim-specific total IgG or IgG1 were detected colorimetrically using anti-mouse IgG Fc-HRP for color development. The absorbance at 450 nm was measured with an ELISA microplate reader (Molecular Devices Inc.) and the optical density was determined.

### 2.6 Immunohistochemistry for fibrosis analysis

Skin and lung tissues were fixed in 10% (v/v) neutral-buffered formalin and embedded in paraffin. Tissue sections (5 µm thick) were stained with hematoxylin and eosin (H&E) and examined using a photomicroscope (Olympus, Tokyo, Japan). Dermal thickness was measured using the IMT iSolution Lite software v10 (IMT iSolution Inc.). Areas of collagen deposition in Masson’s trichrome (MT; Polysciences, Inc.) stained sections were identified using a photomicroscope (Olympus).

Dermal thickness and lung fibrosis severity were scored as described previously (28, 29). Dermal thickness was measured from the top of the granular layer to the junction between the dermis and the subcutaneous fat. Lung fibrosis severity was evaluated using the modified Ashcroft scoring system on H&E- and MT-stained sections, as previously described (30). The fibrosis score was obtained by analyzing skin and lung cross-sections from H&E- and MT-stained slides. Immunohistochemistry was performed using a Vectastain ABC kit (Vector Laboratories) in sections incubated with specific antibodies (anti-smooth muscle antibody [αSMA; Abcam], anti-cell surface Vim [CSV; Abnova], anti-Col1A1, anit-IL-4, and anti- IL-17 [all from Invitrogen]) overnight at 4°C, then with biotinylated secondary antibody for 30 min at RT, followed by streptavidin-peroxidase complex for 30 min at RT. The final color product was developed using diaminobenzidine (DAKO) as the chromogen. Slides were prepared for each mouse (n=12). Immunoassayed tissue sections were examined under a photomicroscope (Olympus). The number of positive cells in one high-power field (magnification 400×) was determined using Image J software, and average cell numbers were calculated for three randomly selected tissue sections.

### 2.7 Statistical analysis

The statistical analyses were performed using Prism, version 10 for Mac (GraphPad). Multiple groups were compared using one-way ANOVA, two-way ANOVA, or the Kruskal-Wallis test; for groups with significant differences, a Bonferroni or Dunn’s *post hoc* test was used to examine the differences. The numerical data of two groups were compared using a nonparametric Mann–Whitney U test (two-tailed). The results are presented as the mean ± standard deviation.

## 3 Results

### 3.1 Production of mouse vimentin

**Figure 1:**
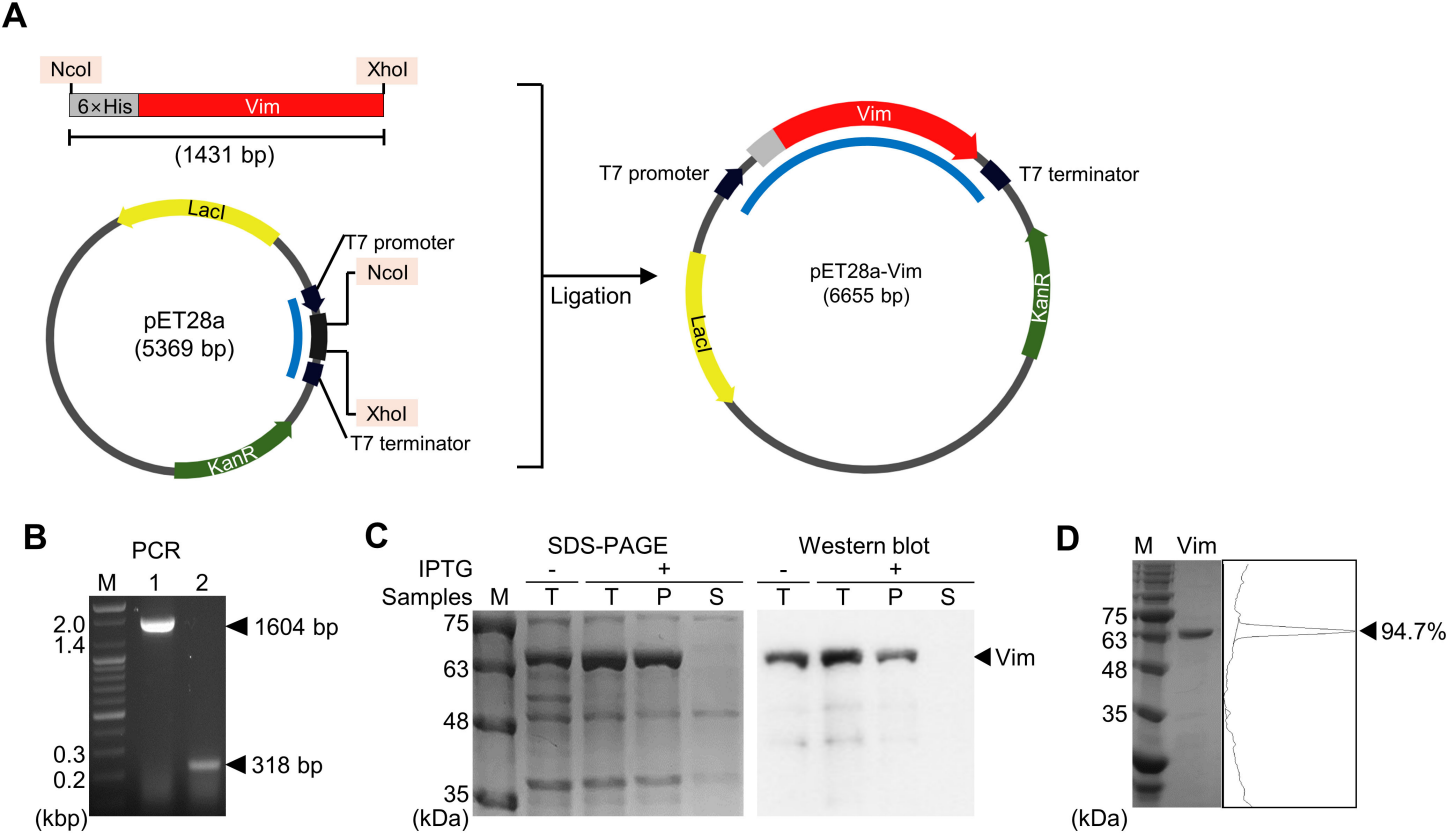
Expression and evaluation of mouse vimentin (Vim). **(A)** Construction of the pET28a-Vim expression vector. The restriction enzyme sites used in cloning are shaded in pink. **(B)** Amplicons generated using a primer set specific to the T7 promoter and terminator, indicated in **(A)** with blue lines. Lane 1: pET28a-Vim, lane 2: pET28a, M: size marker. **(C)** SDS-PAGE and Western blotting analysis of expressed vimentin. T: total lysate, P: pellet, S: supernatant. **(D)** SDS-PAGE analysis of purified vimentin. The band intensity is shown on the right as peaks.

The mouse-Vim-expressing vector pET28a-Vim was generated to produce Vim conjugated with a 6×His tag at the N-terminus (**Figures 1A, B**). Insertion of the *Vim* gene was confirmed by amplifying the region between the T7 promoter and the plasmid terminator using specific primers and then verifying the size of the amplicon (**Figures 1C, D**). As most of the expressed Vim was insoluble, it was solubilized using 6 M urea prior to its purification on a Ni-NTA column. A purity of 94.7% was achieved, and the concentration of the purified Vim protein was 27.7 mg/L.

### 3.2 Frequency of systemic Th2 and Th17 cells is increased in a mouse model of SSC obtained using BLM injection and Vim immunization

**Figure 2:**
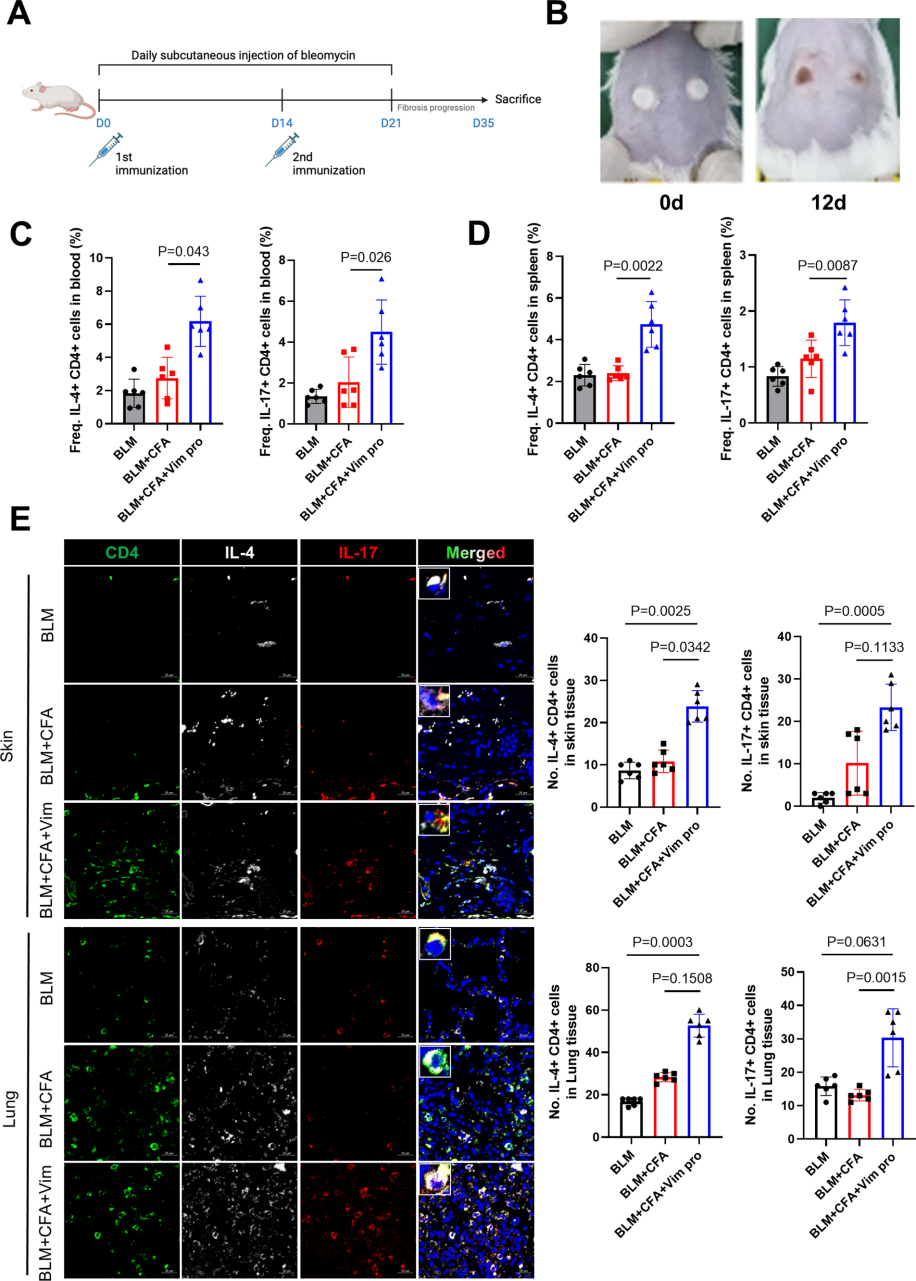
Novel mouse model of systemic sclerosis obtained by combining BLM injection and Vim immunization. **(A)** Protocol for establishing a new mouse model of SSc using BLM injection and Vim immunization (each group n=12). **(B)** Morphology of the dorsal skin in scleroderma mice from vimentin immunized mice at days 0 and 12. White spots on the dorsal surface indicate the sites of intradermal injections of vimentin in a CFA-containing solution **(C)** Frequencies of Th2 (CD4^+^ IL-4^+^) and Th17 (CD4^+^ IL-17^+^) cells in peripheral blood, determined as the proportion of IL-4+ or IL-17+ cells within the CD4+ T lymphocyte population gated from lymphocytes by flow cytometry. **(D)** Frequencies of Th2 and Th17 cells in spleen, calculated in the same way (gated from lymphocytes). **(E)** Immunofluorescence images showing the increased frequencies of Th2 and Th17 cells in the spleens of BCV mice. Bars represent the values from six individual specimens (n=6). The data are presented as the mean ± standard deviation (SD). Scale bars = 20 μm.

The conventional mouse model of SSc obtained by BLM injection is pathologically similar to SSc in humans but it does not completely mimic the disease from an immune cell perspective. Thus, a novel mouse model was established by administering homogenized Vim protein and CFA to the mice during their injection with BLM. As in the conventional model, BLM was injected subcutaneously daily for 21 days. A second group of mice was injected with BLM and a third group with BLM and CFA. In the Vim-treated mice, homogenized Vim protein was mixed with CFA at a 1:1 ratio and administered twice through intradermal injections separated by 2 weeks (**Figures 2A, B**). Morphological examination of the dorsal skin in scleroderma mice from vimentin immunized mice revealed changes at days 0 and 12. The white spots visible on the dorsal surface correspond to intradermal injection sites of vimentin in a CFA-containing solution (**Figure 2B**). All mice were euthanized 35 days after the first BLM injection, and the frequency of helper T cell subsets was examined in the blood and spleen. Both Th2 and Th17 cell frequencies (as a percentage of CD4+ T cells) increased remarkably in BLM-treated Vim-injected mice (BC+Vim; BCV, **Figures 2C, D**). An analysis of the immune cells in skin and lung tissues showed increases in infiltrated Th2 and Th17 cells in BCV mice (**Figure 2E**). These results demonstrated that the combined injection of BLM and Vim+CFA increases Th2 and Th17 cell numbers, not only systemically but also within SSc lesions.

### 3.3 Vimentin immunization leads to B cell activation and autoreactivity in BCV mice

**Figure 3:**
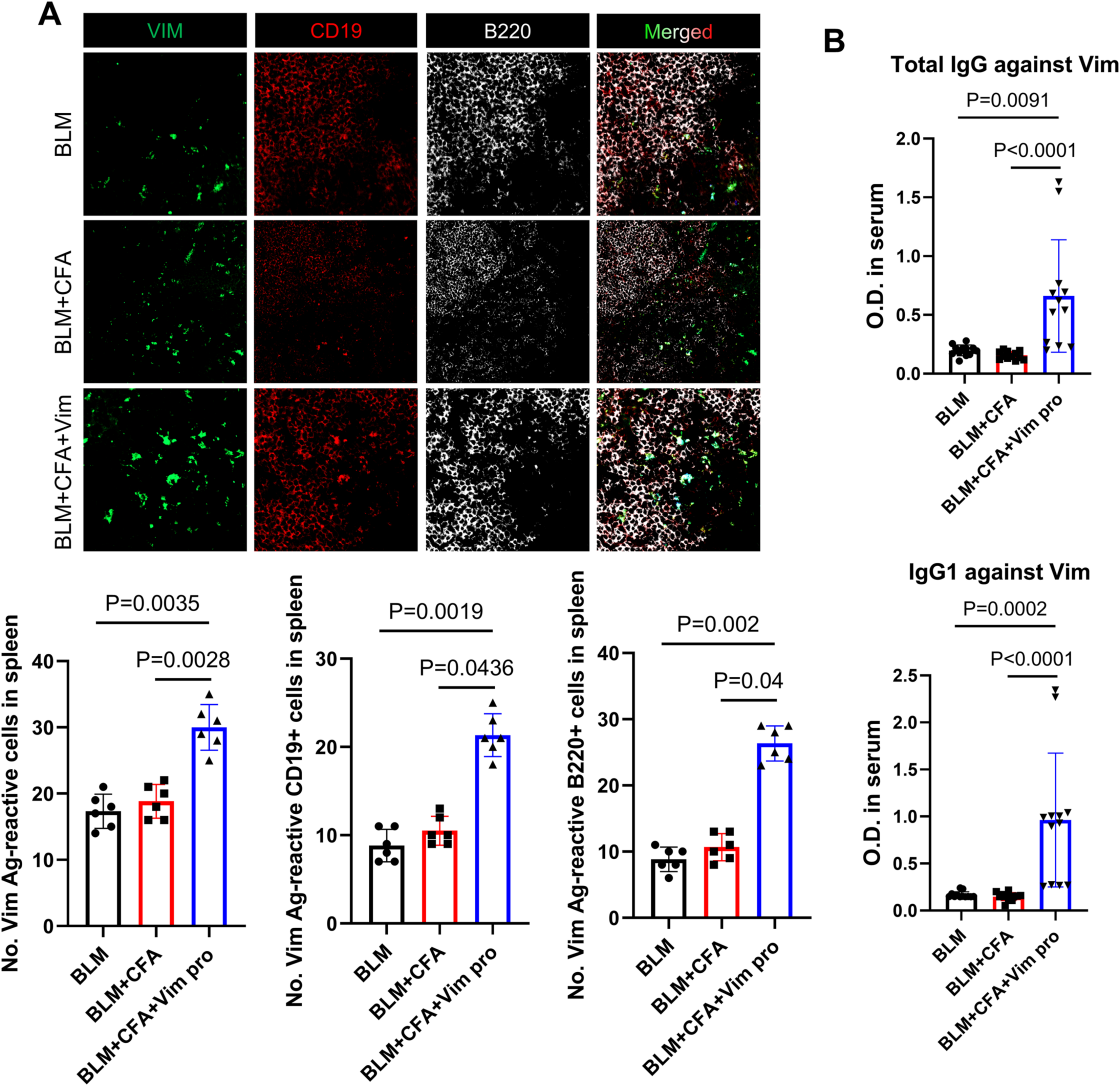
Increased B cell activation and autoantibody production in a novel mouse model of SSc. **(A)** Immunofluorescence images showing the reaction of spleen tissues with FITC-conjugated soluble Vim, PE-conjugated anti-CD19 antibodies, and Alexa Fluor 647-conjugated anti-B220 antibodies in each mouse model of SSc. Bars represent the values from six individual specimens (n=6). **(B)** Serum levels of total anti-Vim IgG and anti-Vim IgG1 in each mouse model (n=12) as detected by ELISA. The data are presented as the mean ± SD.

To investigate whether the administration of BLM and Vim activates B cells autoreactive for Vim, autoreactive antibody titers and the number of spleen B cells were determined as follows: FITC-tagged Vim protein was reacted with spleen tissues from the three groups of mice. The number of B cells and the frequency of autoreactive B cells in the spleen were assessed by staining with anti-B220 and anti-CD19 antibodies. The results showed increases in the number of B cells and in the number of Vim-positive cells in the spleens of the BCV group (**Figure 3A**). The serum concentration of anti-Vim antibodies was measured to determine whether these B cells produce and secrete autoantibodies against Vim (**Figure 3B**). The concentrations of total IgG and IgG1 against Vim were significantly higher in BCV mice. These results demonstrated that, in the novel SSc animal model, Vim autoantibodies are produced.

### 3.4 Combined injection of BLM and Vim increases symptom severity in SSc

**Figure 4:**
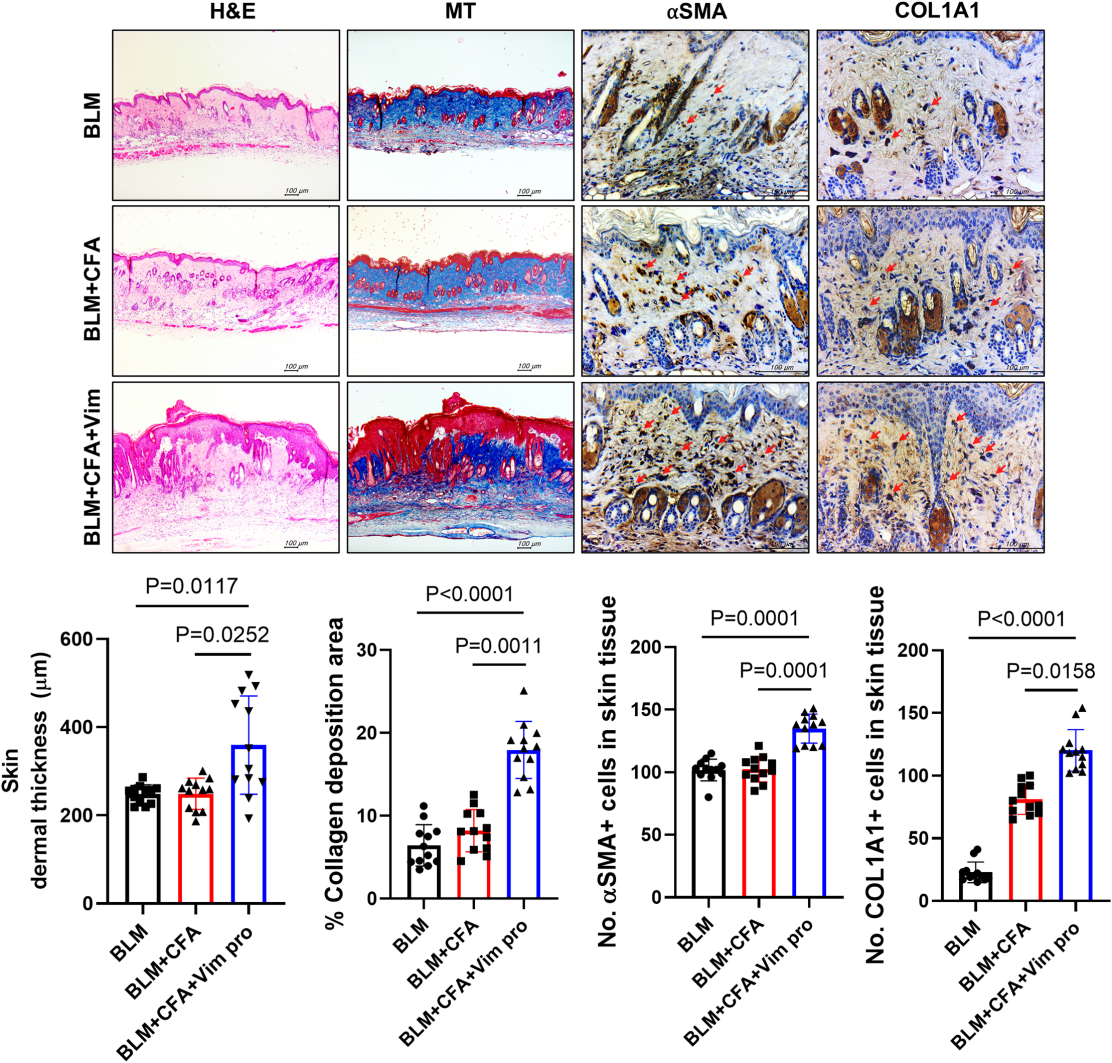
Mice injected with both BLM and Vim exhibit more severe pathological symptoms of scleroderma than mice injected with BLM alone. Skin thickness (n=12) and collagen deposition were assessed based on H&E and MT staining; αSMA and Col1A1 levels were detected by immunohistochemistry. Two tissue slides were prepared for each mouse. The number of positive cells in one high-power field (magnification 400×) was determined using Image J software, and average cell numbers were calculated for three randomly selected tissue sections. The data are presented as the mean ± SD. Scale bars = 100 μm.

The pathological changes in the skin tissues of the mice were investigated by H&E, MT, and immunohistochemistry staining. In the H&E- and MT-stained sections, greater skin thickness and larger areas of collagen deposition were observed in the BCV group, indicating more severe skin sclerosis in these mice. In addition, the expression levels of the fibrosis markers αSMA and Col1A1 were significantly higher in the BCV group than in the BLM and BLM+CFA (BC) mice (**Figure 4**). Thus, immunization with Vim protein provides an animal model of SSc that more closely resembles SSc in humans than the model based on BLM alone.

### 3.5 Combined injection of BLM and Vim induces severe pathological symptoms in the lung

**Figure 5:**
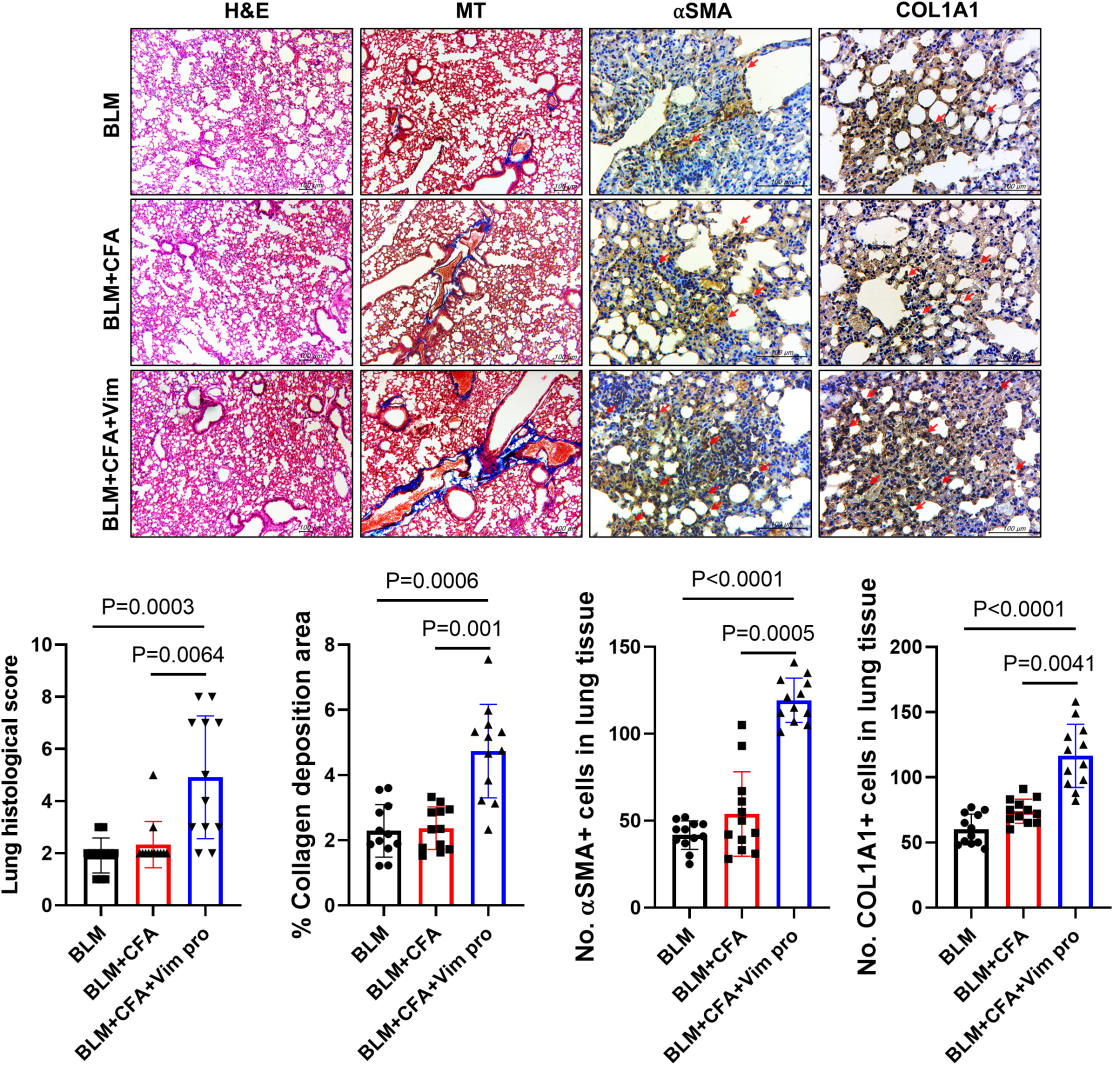
Mice injected with both BLM and Vim exhibit more severe lung sclerosis than mice injected with BLM alone. Tissue thickness and collagen deposition in lung tissues (n=12) were assessed based on H&E and MT staining (images acquired at 100× magnification). The levels of αSMA and Col1A1 were detected via immunohistochemistry (IHC images acquired at 400× magnification). Two tissue slides were prepared for each mouse. The number of positive cells in one high-power field was determined using Image J software, and average cell numbers were calculated for three randomly selected tissue sections. The data are presented as the mean ± SD. Scale bars = 100 μm.

Similar to human SSc, skin and lung sclerosis were observed in the mouse models. In Vim-treated mice, lung tissue was examined to determine whether Vim immunization has further effects on lung fibrosis. Pathological symptoms were evaluated based on H&E, MT, and immunohistochemistry staining. In the lung tissues of BCV group, the lung histological score, collagen deposition areas, and expression levels of αSMA and Col1A1 were higher than in the control mice (**Figure 5**). These results demonstrate the validity of our novel mouse model in studies of lung fibrosis, such as occurs in SSc.

### 3.6 Pro-inflammatory cytokines are upregulated in skin and lung tissues of BCV mice

**Figure 6:**
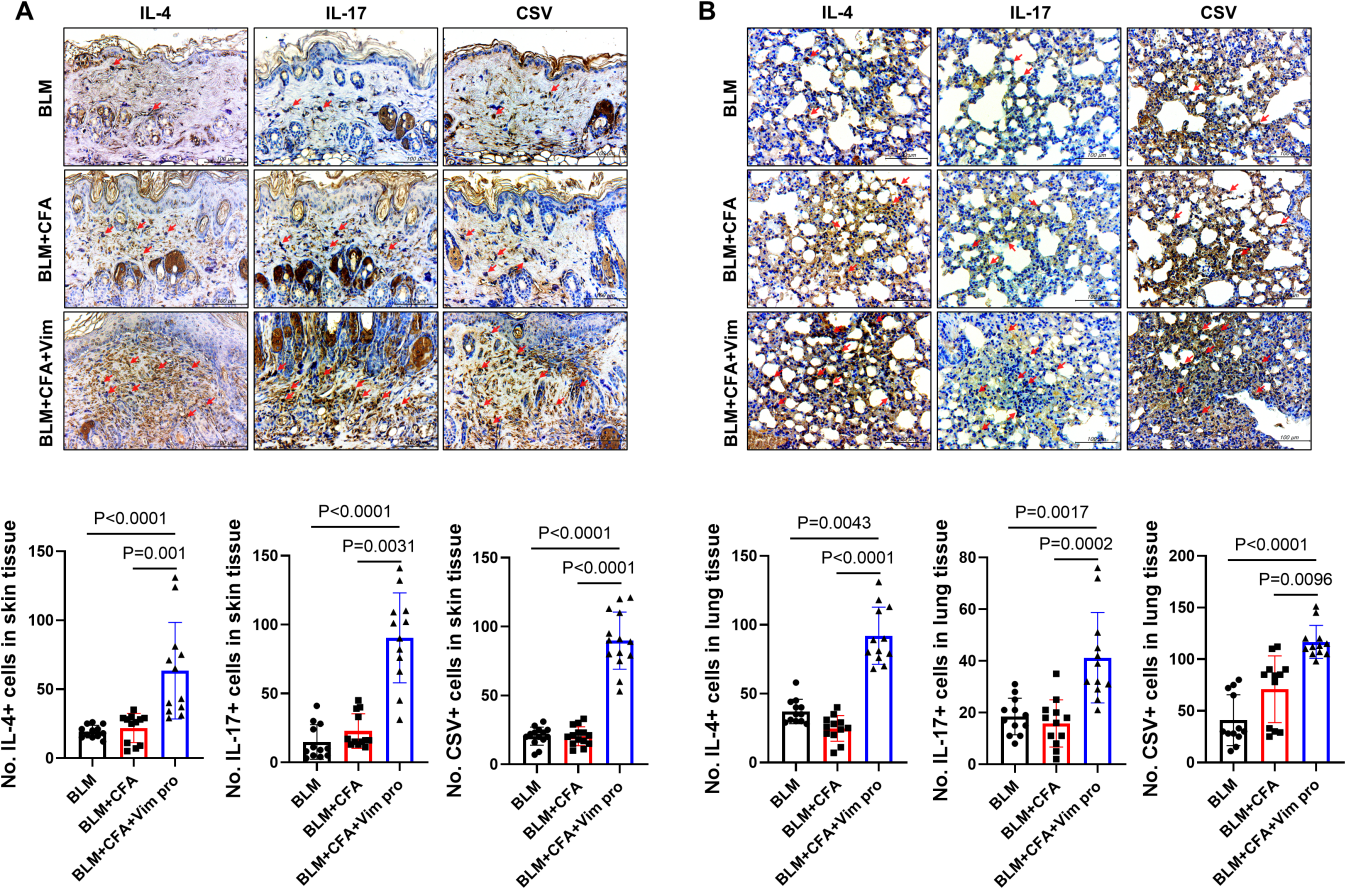
Activated pro-inflammatory cytokines in the fibrosing skin lesions of mice injected with both BLM and Vim. **(A, B)** The expression of IL-4, IL-17, and cell surface vimentin (CSV) in skin **(A)** and lung tissues **(B)** was determined via immunohistochemistry (n=12). Two tissue slides were prepared for each mouse. The number of positive cells in one high-power field was determined using Image J software, and average cell numbers were calculated for three randomly selected tissue sections. The data are presented as the mean ± SD. Scale bars = 100 μm. The English in this document has been checked by at least two professional editors, both native speakers of English. For a certificate, please see: http://www.textcheck.com/certificate/7CUcgu.

Whether the newly established mouse model of SSc was accompanied by immunological changes was investigated by immunohistochemically staining skin and lung tissues for pro-inflammatory cytokines and CSV. The levels of the pro-inflammatory cytokines IL-4 and IL-17 in skin tissue were significantly higher in BCV mice than in BLM or BC mice (**Figures 6A, B**). By contrast, their expression did not significantly differ between the latter two groups. CSV expression was also significantly increased in both the skin and the lung tissues of BCV mice. These results imply the presence of activated immune cells in BCV mice, thus suggesting that this novel model is a more effective animal model for studies of the autoimmune features of SSc.

## 4 Discussion

SSc is an autoimmune disease that causes thickening of the skin and lung tissues, tissue stiffness, exhaustion, and abnormal blood flow (2, 31). Although its pathogenesis is thus far unclear, pro-inflammatory cytokines, immune cells, and the tissue microenvironment are contributing factors (31, 32). BLM-treated mice have long served as an animal model of pulmonary fibrosis (33) and in the development of treatments for SSc (18). However, unlike other autoimmune diseases, such as rheumatoid arthritis or multiple sclerosis, no animal model mimics the immunological changes in SSc (21).

As a major component of intermediate filaments in human dermal fibroblasts (34), Vim is required for a broad range of vital cell functions, such as cell motility, chemotactic migration, and wound healing (35, 36). Vim modification has been implicated in the loss of fibroblast contractility that occurs during aging, as it leads to the structural breakdown of the intermediate filament system (34). Previous studies have demonstrated high vimentin expression in animal models of SSc (37). Notably, serum citrullinated vimentin (VICM) levels serve as a biomarker of disease activity in systemic sclerosis, showing significantly elevated concentrations in patients with rapid disease progression compared to healthy controls (24). Another study found that Vim is highly expressed in the endothelial cells of patients with progressive SSc; electron microscopy showed microvessels with occluded lumina due to the presence of swollen endothelial cells, the cytoplasm of which was filled with Vim type filaments (25). These observations suggest that the combined use of BLM and immunization against Vim protein would provide a novel animal model of SSc that reflects the immunological changes in the disease.

To generate robust autoantibodies against vimentin (Vim) and establish autoreactive T and B cell responses, mice received primary and booster immunizations with CFA and Vim protein homogenate at day 0 and day 14, following a standard prime-boost protocol to maximize immune activation. Concurrently, beginning on day 1, BLM was injected daily for 21 days to induce fibrotic changes. This approach combining vimentin immunization with bleomycin treatment was designed to enhance both autoimmune and fibrotic components of the disease model. Conventional BLM-induced SSc mice and background control (BC) mice were established as comparison groups. In the newly developed vimentin-immunized SSc model, the levels of pro-inflammatory cytokines, autoantibodies, and activated immune cells in the analyzed tissues were significantly elevated compared to controls, demonstrating successful induction of both autoimmune and fibrotic pathology.

Systemic immunological changes in the BCV mice were confirmed based on the frequency of Th2 and Th17 cells in the spleen and blood as well as the levels of the pro-inflammatory cytokines IL-4 and IL-17. BCV mice also had elevated concentrations of Vim autoantibodies. These results suggest that Vim immunization increases not only helper T cell numbers but also the number of autoreactive B cells. The frequency of B cells in the spleen and the ability of these cells to bind Vim antigen were examined as well. In the new model, the number of B cells recognizing soluble Vim was significantly increased in spleen tissue. Therefore, in contrast to the conventional mouse model of SSc, our novel model includes systemic immune activation, and particularly the activation of autoreactive B cells. It is therefore a suitable model for studying progressive SSc.

The pathological changes in the skin tissues of the mice were assessed in H&E- and MT-stained sections and via immunohistochemistry. In BCV mice, skin thickness and the area of collagen deposition were increased, as were the expression levels of αSMA and Col1A1, markers of fibrosis. Because SSc in humans and in mouse models causes fibrosis not only in the skin but also in the lungs, the pathological changes in lung tissues were similarly examined. As in the skin, the expression levels of αSMA, ColA1, and pro-inflammatory cytokines were increased.

We developed a novel animal model of progressive SSc, established by simultaneously injecting mice with BLM and Vim. In contrast to the conventional models of SSc, obtained by BLM injection alone, the new model features activated immune cells and the increased production of autoantibodies against Vim. This new model can be used in studies of the pathogenesis and treatment of severe SSc.

## 5 Conclusion

The vimentin immunization model of systemic sclerosis exhibited accelerated skin and lung fibrosis compared to the bleomycin (BLM)-induced model, along with increased activation of Th2 and Th17 cells and enhanced autoantibody production against vimentin. These findings suggest that vimentin antigenic stimulation leads to more severe pathology and immune cell activation than does BLM, and better recapitulates immune cell activation. Thus, the vimentin immunization model may provide a valuable tool for developing new therapeutic strategies for systemic sclerosis.

## Data Availability

The raw data supporting the conclusions of this article will be made available by the authors, without undue reservation.
