# Supplementary material for: Vimentin immunization induces TH2/TH17 cell activation and autoantibody production in a novel mouse model of bleomycin induced systemic sclerosis
Source: Front Immunol. 2025 Nov 7;16:1639601. doi: 10.3389/fimmu.2025.1639601 (PMC12634585; doi:10.3389/fimmu.2025.1639601)
Supplement: Supplementary file 1 [file Table1.docx]

# Supplementary Data

**Full-length nucleotide sequence of the inserted 6×His-tagged Vim**

catcatcatcatcatcacAGCAGATCTGTGTCGAGCTCATCGTACCGTAGAATGTTTGGTGGCTCGGGAACGTCTAGTCGCCCGAGCAGCAACCGCAGCTACGTAACTACGTCAACGCGCACCTATTCACTTGGTAGCGCCTTGAGACCCTCCACTTCGAGATCCTTATACTCATCCTCGCCCGGGGGGGCATACGTCACCAGAAGCAGCGCAGTCCGGTTGAGATCTTCAGTTCCAGGTGTTCGCCTGCTGCAGGATTCAGTTGACTTTTCGTTAGCGGACGCGATTAATACAGAGTTCAAAAACACGAGAACTAATGAAAAAGTAGAACTGCAGGAGCTTAACGATCGGTTCGCTAACTATATAGACAAGGTTAGATTCCTTGAACAACAAAATAAAATTCTGTTAGCCGAACTTGAACAGTTAAAAGGCCAGGGAAAGAGCCGTCTGGGAGACTTATATGAAGAAGAGATGCGGGAGTTACGCAGACAGGTCGACCAGCTTACTAATGATAAAGCTAGAGTGGAGGTGGAACGTGACAATTTGGCGGAAGATATAATGAGACTTAGAGAGAAATTGCAAGAGGAGATGCTGCAACGGGAAGAAGCGGAGAGCACATTGCAATCGTTTCGGCAGGATGTAGACAACGCTTCCCTGGCGCGTTTAGACCTTGAGAGAAAAGTGGAATCCCTTCAGGAAGAGATTGCATTTTTGAAAAAGCTGCATGACGAAGAAATTCAAGAGCTGCAGGCTCAAATTCAGGAACAGCATGTACAAATTGACGTCGACGTTTCGAAACCCGACCTTACAGCAGCTCTTCGTGACGTTCGCCAACAATATGAGAGTGTTGCTGCGAAGAATCTGCAGGAAGCAGAAGAATGGTATAAATCAAAGTTTGCGGATTTAAGCGAGGCAGCAAACAGAAACAATGACGCGCTGCGCCAGGCAAAGCAAGAAAGCAATGAGTATCGCCGCCAAGTCCAAAGCCTGACTTGCGAAGTAGATGCGTTAAAAGGGACTAACGAATCACTGGAACGCCAGATGCGGGAGATGGAGGAAAATTTCGCTCTGGAGGCGGCAAACTATCAAGACACAATTGGTCGCCTGCAAGACGAAATTCAAAATATGAAAGAAGAAATGGCGCGGCATCTGCGTGAATATCAGGATTTACTTAACGTCAAGATGGCGTTGGACATAGAAATTGCTACTTACCGTAAGCTTTTAGAAGGTGAAGAGAGTAGAATATCGTTGCCATTACCTACCTTTAGCTCGCTGAACCTTCGCGAGACTAACCTTGAAAGCCTTCCACTGGTGGACACTCATTCCAAAAGAACGCTTCTGATAAAGACCGTAGAAACACGCGACGGTCAAGTCATCAATGAGACCAGTCAACATCACGACGACCTGGAATAA
